# Research on the Synthesis of Zinc–Ammonium Phosphate Using Galvanic Waste Sludge as a Source of Zinc

**DOI:** 10.3390/ma17071690

**Published:** 2024-04-07

**Authors:** Claudia Mona Morgovan, Anda Ioana Gratiela Petrehele, Gabriela Elena Badea, Alexandrina Fodor, Monica Toderaș, Eleonora Marian

**Affiliations:** 1 Department of Chemistry, Faculty of Informatics and Sciences, University of Oradea, 1 University Street, 410087 Oradea, Romania; cmorgovan@yahoo.com (C.M.M.);; 2 Department of Physics, Faculty of Informatics and Sciences, University of Oradea, 1 University Street, 410087 Oradea, Romania; 3 Department of Pharmacy, Faculty of Medicine and Pharmacy, University of Oradea, 29 N. Jiga Street, 410028 Oradea, Romania; marian_eleonora@yahoo.com

**Keywords:** waste sludge, microelement fertilizer, zinc–ammonium phosphate

## Abstract

This paper presents the extraction of zinc ions from waste resulting from the galvanic industry, such as sludge from acid zinc electroplating baths, and their revaluation in mineral fertilizer as zinc–ammonium phosphate. The purpose of this work is to extract zinc ions from the sludge that forms directly in the zinc bath, which can only contain zinc and small amounts of iron, to revalorize zinc into the form of zinc–ammonium phosphate. The process of obtaining zinc–ammonium phosphate is presented using waste sludge from the galvanic industry. In order to obtain zinc–ammonium phosphate, the solution resulting from the dissolution of the sludge with a 20% hydrochloric acid solution was used in reaction with diammonium phosphate and a 25% ammonia solution. After the chemical analysis of the obtained products, zinc–ammonium phosphate was characterized using X-ray powder diffraction, infrared FT-IR spectroscopy and electronic microscopy (SEM) analysis. The results obtained indicate a promising approach to sustainable resource utilization in the production of zinc–ammonium phosphate.

## 1. Introduction

Environmental protection and manufacturing technology are interconnected. Today, there is a global trend towards improving existing technologies and promoting new technologies, so-called clean or sustainable technologies, that do not generate waste or have the possibility of waste recycling. The goal of every technology is to create useful products, but at the same time, various types of waste are produced. The development and modernization of the mineral fertilizer industry receive special attention all over the world. Agriculture requires concentrated, complex, solid and liquid fertilizers, as well as fertilizers with microelements. This work focuses on the extraction of zinc ions from waste and their utilization (revalorization) in the form of zinc–ammonium phosphate [1].

Zinc–ammonium phosphate is primarily used as a slow-release chemical fertilizer of macro elements (nitrogen, phosphorus) and microelement Zn. Zinc–ammonium phosphate is widely used as a corrosion inhibitor, particularly in coatings for metal surfaces. Its ability to form a protective layer on metal substrates helps prevent corrosion, making it valuable in industries where protection against rust and degradation is critical. Due to its fire-retardant properties, zinc–ammonium phosphate is utilized in various applications, including flame-retardant coatings for textiles and polymers. The compound releases water when exposed to heat, creating a cooling effect and inhibiting the spread of flames. In agriculture, zinc–ammonium phosphate serves as a phosphorus and zinc fertilizer additive. It provides essential nutrients to plants, promoting growth and enhancing crop yield. Its controlled release properties make it a valuable component in fertilizer formulations. Zinc–ammonium phosphate finds applications in the ceramics and glass industries as a fluxing agent. It facilitates the melting of raw materials during the production of ceramics and glass, aiding in the formation of a homogenous and durable final product. The compound is employed in water treatment processes to inhibit scale formation and control corrosion in water systems. Its effectiveness in preventing mineral deposits makes it a valuable additive in cooling water treatment. In certain medical formulations, zinc–ammonium phosphate may be used for its antimicrobial properties. Research is ongoing to explore its potential in medical coatings and materials. While zinc–ammonium phosphate has various industrial applications, its use should be mindful of environmental impacts. Proper disposal and management practices are essential to minimize any potential ecological effects [2,3,4,5,6,7,8,9,10].

Zinc’s role in plants is to help them produce chlorophyll. Zinc, an essential microelement, plays a pivotal role in the growth and development of plants. Despite being required in relatively small quantities, zinc is indispensable for various physiological processes within plants. Zinc is classified as a microelement because plants require it in smaller quantities compared to primary or macro elements like nitrogen, phosphorus and potassium. However, its significance is no less crucial for the well-being of plants. Zinc is a key component in numerous enzymes involved in various metabolic pathways. It plays a vital role in the activation of enzymes that participate in processes such as photosynthesis, which is essential for the production of energy and plant growth. Zinc is an important component of DNA synthesis and cell division. It helps regulate the expression of genes involved in these processes, aids in the formation of new tissue and ensures proper growth and development. The synthesis of proteins, a fundamental aspect of plant growth, is influenced by zinc. Additionally, zinc is involved in nitrogen metabolism, aiding in the efficient utilization of nitrogen by plants. Zinc plays a crucial role in root development and architecture. Adequate zinc levels enhance the plant’s ability to absorb water and essential nutrients from the soil. This, in turn, contributes to overall plant vigor. Zinc contributes to the plant’s ability to withstand stress conditions, including environmental stress and pathogen attacks. It is involved in the synthesis of compounds that enhance the plant’s defense mechanisms, bolstering its immunity. Zinc influences flowering and seed formation processes. It is particularly important for the development of reproductive structures, ensuring successful pollination and the production of viable seeds for the next generation of plants. In agriculture, ensuring an optimal supply of zinc is crucial for maximizing crop yield and quality. Zinc fertilizers are commonly used to address zinc deficiencies in soils, ensuring that plants have access to this vital microelement [11].

Zinc deficiency can cause leaf discoloration known as chlorosis, which causes the leaves to turn yellow. Chlorosis caused by zinc deficiency usually affects the base of leaves near the stem. Chlorosis first appears on the lower leaves and then gradually moves upward. In severe cases, the upper leaves will turn yellow and the lower leaves will turn brown or purple and die. Phosphorus compounds play an important role in plant growth because without this element or in insufficient amounts plants cannot grow properly and seeds will not form [12].

The supplement of phosphorus necessary for poor soil may be assimilated by adding ammonium phosphates and metal–ammonium phosphates, which also represent a source of microelements. Zinc–ammonium phosphate is important as a primary compound fertilizer, with zinc as a microelement and as an industrial phosphate. Zinc–ammonium phosphate contains zinc, which is related to the macro elements nitrogen and phosphorus and improves the efficiency of the plant uptake process of fertilizers [1].

Waste sludge from industry or urban wastewater treatment plants is a source of revalorization of metals and other useful substances [13,14,15,16,17,18,19,20,21]. Galvanic sludges, generated as a byproduct of electroplating processes, contain valuable metals such as zinc, copper, nickel and chromium. Effectively recovering these metals not only conserves valuable resources but also mitigates their environmental impact. Understanding the composition of galvanic sludges is crucial for effective recovery. Analyzing the sludges for metal content, impurities and physical properties provides essential insights into the most suitable recovery methods. Leaching is a common technique used to extract metals from sludges. Acidic or alkaline leaching solutions are applied to dissolve metals selectively. This process can be tailored to target specific metals based on their solubility, allowing for a more controlled extraction. Solvent extraction involves using organic solvents to selectively extract metals from leach solutions. This method is particularly effective for separating metals with similar chemical properties. The extracted metals can then be further purified for reuse. Electrowinning is a widely employed electrochemical process where metals are plated onto electrodes. After leaching, the metal-rich solution is subjected to electrowinning, resulting in the deposition of pure metals. This method is efficient for recovering metals like copper and nickel. Ion exchange resins can selectively adsorb metal ions from leach solutions [22]. This method offers a high degree of specificity and can be employed to target specific metals for recovery. Ion exchange is often used in conjunction with other processes for comprehensive metal extraction. Metal precipitation involves adjusting the pH of the leach solution to induce the precipitation of metal hydroxides or sulfides. Once precipitated, the metals can be separated and further processed. This method is particularly effective for recovering metals like chromium. While metal recovery is essential, it is equally crucial to address environmental concerns associated with galvanic sludges. Implementing eco-friendly methods and ensuring proper disposal of remaining materials are vital components of a sustainable recovery process. Assessing the economic viability of metal recovery is crucial for industry adoption. Factors such as market prices, extraction costs and regulatory compliance play a significant role in determining the feasibility of implementing these recovery methods.

To obtain zinc–ammonium phosphate, industrial waste or residual solutions can be used as zinc sources [1].

## 2. Materials and Methods

The galvanic industry is one of the most important industries in the world, especially for the automotive industry, because the coatings of steel parts with thin layers of other metals give them better corrosion resistance, firstly, as well as superior mechanical and chemical properties. However, this industry generates residues that contain large amounts of heavy metals. In the case of acid zinc coatings, the main operations are mechanical part preparation, alkaline degreasing, pickling in acids, zinc plating in acid electrolyte, passivation in chromate solutions and washes between all these operations.

Wastewater is neutralized in treatment plants before being discharged into the sewers. Thus, purification is performed, consisting of reducing Cr^6+^ to Cr^3+^, the precipitation of metal ions (Zn, Cr, Fe) in the form of hydroxides, sedimentation of suspensions, recirculation/evacuation of water, sludge treatment and evacuation. The sludge formed by neutralizing wastewater is usually stored on specially designed land, which presents many disadvantages primarily due to its environmental impact contributing to the increase of the land areas covered with hazardous materials, as well as from an economic point of view because the recovery possibilities of heavy metals for their reuse are not taken into account.

Because this final resulting sludge contains a varied mixture of metals, including chromium, which is harmful to plants, the purpose of this work is to extract zinc ions from the sludge that forms directly in the zinc bath, which can only contain zinc and small amounts of iron, to revalorize zinc into the form of zinc–ammonium phosphate [1,23,24].

The sludge samples were taken in equal quantities from three acid zinc galvanizing baths on the same electroplating stream of a company producing parts for the automotive industry. The samples were mixed, homogenized, filtered under vacuum and dried in the oven at 110 °C. This sludge was first analyzed to determine its chemical composition. To be brought into the solution, it was mineralized, and a well-determined amount of dry sludge (3 g) was treated hot with a mixture of HNO_3_ and HCl (15 mL) in a ratio of 3:1. After the sample was dry, it was brought to a 100 mL flask and the concentration of metals (Zn, Fe, Cu, K and Na) was determined.

The zinc extraction from the waste was made with solutions of hydrochloric acid at different concentrations (10%, 20%, 30%, 35%) by mechanical stirring for 20 min. A well-defined volume of acid (20 mL) and a stoichiometric quantity of sludge were used.

After stirring, the reaction mass was filtered, and the concentration of zinc was determined from the solution. After establishing the optimal conditions for zinc extraction, the solution was treated with a stoichiometric quantity of diammonium phosphate and then neutralized with a 25% ammonia solution to reevaluate the zinc ions as zinc–ammonium phosphate [1].

The pH of the reaction mass was determined at different NH_3_:Zn^2+^ ratios, taking into account the added ammonia. The precipitated product, separated by filtration, was dried in the oven at 70 °C [1,23,24].

All reagents used were purchased from Merck (Darmstadt, Germany), and the solutions were prepared using deionized water.

In the filtrate, the residual zinc content was determined, and the solid products were subjected to a complex study to determine their physicochemical properties: chemical composition, RX diffraction analysis, FT-IR spectroscopic analysis, thermogravimetric analysis and electron microscopy (SEM).

The concentration of metals in the sludge, the zinc content of the filtrate and the finished product were determined with atomic absorption spectrophotometry using a VARIAN SPECTR AA 110 spectrophotometer (Varian Inc., Palo Alto, CA, USA) [1,23,24].

A Mettler Toledo Switzerland pH/ion (Mettler Toledo GmbH, Greifensee, Schweiz) analyzer was used to determine the pH of the reaction mass.

The ammoniacal nitrogen and phosphorus pentoxide contents of the finite product were determined using a VARIAN CARY 50 Probe spectrophotometer (Varian Inc., Palo Alto, CA, USA).

The FT-IR spectroscopic investigations of the obtained products were made with a JASCO 6100 spectrometer (Jasco Inc., Easton, MD, USA) in the spectral range of 4000–400 cm^−1^ with a resolution of 2 cm^−1^ using the KBr pellet technique. X-ray diffraction was measured with a Shimadzu 6000 diffractometer (Shimadzu, Kyoto, Japan) using CuKα radiation [25].

The thermogravimetric analyses were performed with the Perkin Elmer TGA 7 thermobalance (Perkin Elmer Inc., Shelton, CT, USA), which uses Perkin Elmer Thermal Analysis Software Version 2.00. A standard oven was used, allowing heating from ambient temperature up to 1000 °C, as well as a Chromel–Alumel thermocouple and platinum crucibles, allowing for rapid heating and cooling at 200 °C/min. The temperature range studied was 65–500 °C. Thermal determinations were performed in a dynamic nitrogen atmosphere with a current of 20–35 mL/min, and the warm-up rate β was 7 °C/min [25].

The microscopic examinations were performed under a SEM scanning electron microscope type Jeol 5600 LV (Jeol, Tokyo, Japan), equipped with an Oxford Instrument X-ray spectrometer (Oxford Instruments X-ray Technology, Scotts Valley, CA, USA), which presents the following characteristics: resolution of 3.5 nm with secondary electrons, increasing 300,000 times; methods of work in high vacuum (HV) and low vacuum (LV); local quantitative chemical analyses based on the characteristic spectrum (EDS) of X-rays in the elements between boron and uranium, with a detection limit of 0.01%; and analysis soft INCA Version 7.00 [26].

The experimental data were processed using the “Origin 5.0” and “Table Curve 3D” programs.

## 3. Results and Discussion

### 3.1. Studies on Zinc Waste

#### 3.1.1. Composition

Experimental data on the composition of the sludge used in acid zinc galvanizing baths are presented in Table 1.

From the experimental data, it is observed that the sludge has a high content of zinc; small amounts of iron; and traces of copper, sodium and potassium. Additionally, chromium was below the detection limit of the apparatus (bdl). Since it is proposed to use this sludge for the synthesis of chemical fertilizer, the reduced amounts of iron, copper and potassium are not inconvenient because they are not toxic to plants.

#### 3.1.2. Dissolution

**Figure 1:**
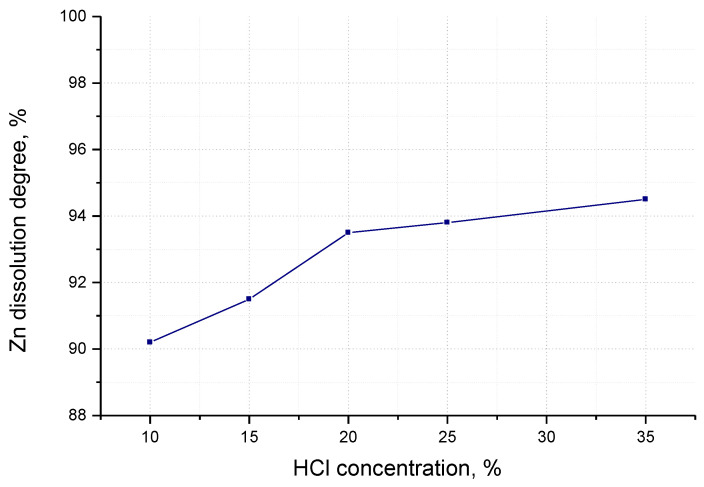
Dependence of the dissolution degree of zinc in the sludge in the concentration of the hydrochloric acid solution.

Experimental data on sludge dissolving in hydrochloric acid solutions of different concentrations are given in Figure 1.

The experimental data show that the dissolution degree of zinc in the sludge increased as the acid concentration is increased to 20%. For acid concentration values of 25%, 30% and 35% or concentrated acid, respectively, the increase is very slow. The differences in the dissolution degree of zinc are not very large depending on the acid concentrations used, and the values for the 20% concentration acid and the 35% concentration acid are very close so that the solution obtained by dissolving the sludge in a hydrochloric acid solution with a concentration of 20% was used further.

### 3.2. Studies Regarding the Separation Process of Zinc from Solution

#### 3.2.1. pH of the Reaction Mass

Zinc–ammonium phosphate is obtained by processing zinc chloride solutions with diammonium phosphate and ammonia according to the reaction:

ZnCl_2_ + (NH_4_)_2_HPO_4_ + NH_3_ = ZnNH_4_PO_4_ + 2NH_4_Cl (1)

The process of obtaining zinc–ammonium phosphate is carried out in two phases. In the first phase, the zinc chloride solution is treated with diammonium phosphate (solid or saturated solution), and in the second phase, the reaction mass obtained is further neutralized with ammonia. The neutralization phase control parameter is the pH of the reaction mass.

In order to obtain zinc–ammonium phosphate, the solution resulting from the dissolution of the sludge with a 20% hydrochloric acid solution was used. For a well-determined volume of zinc chloride solution (75 mL), the stoichiometric amount of diammonium phosphate was calculated, and then 25% ammonia solution was added, following the variation of the pH of the reaction mass according to the ratio NH_3_:Zn^2+^ (mass and molar) [1,23,24].

**Figure 2:**
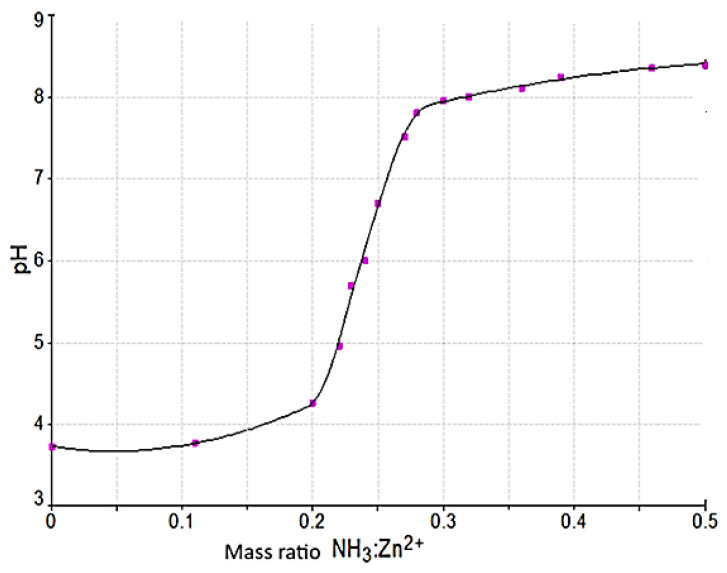
Variation in the pH of the reaction mass as a function of the ratio of NH_3_:Zn^2+^ (mass) for a solution with 265 g/L Zn^2+^ at 25 °C.

Experimental data on the pH variation of the reaction mass as a function of the NH_3_:Zn^2+^ ratio (mass and molar) is presented in Figure 2.

When ammonia is added to the system, the pH of the reaction mass varies with the increase in the amount of ammonia introduced, respectively, with the ratio of NH_3_:Zn^2+^ of the reaction mass. There is a well-defined dependence between the pH of the reaction mass and the mass ratio of NH_3_:Zn^2+^.

From the experimental data, it is observed that the pH of the reaction mass increases slowly to the value of 3.96; in the range of 3.96–7.95, it shows a sudden increase; and above this value, it has a slow growth again. The experimental research has shown that the shape of the curve is not influenced by the zinc concentration of the solutions.

At pH > 8, the increase in the ratio of NH_3_:Zn^2+^ causes the partial ammonia pressure to rise above the system, thus resulting in significant losses of ammonia without essentially influencing the pH value of the reaction mass. Probably, its value is influenced by the NH_4_Cl-NH_3_ buffer system in the solution [1,22].

#### 3.2.2. The Separation Degree of Zinc from Solution

In order to establish the optimal conditions for the process of obtaining zinc–ammonium phosphate from zinc chloride solutions, by processing with diammonium phosphate and ammonia, the influence of certain factors (final pH of the reaction mass, molar ratio NH_3_:Zn^2+^, concentration of zinc chloride solution, temperature, process duration) was studied on the separation degree of zinc.

- pH of the reaction mass.

**Figure 3:**
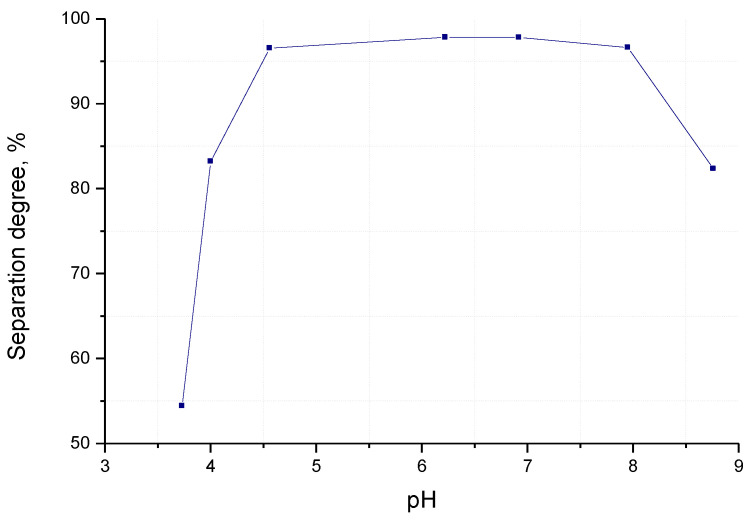
Dependence of the separation degree of zinc (α) from the solution of pH of the reaction mass for a solution of 265 g/L Zn^2+^, at 25 °C.

The experimental data on the influence of the pH of the reaction mass on the separation degree of zinc from the solution is shown in Figure 3.

The separation degree of zinc from the solution after the addition of the diammonium phosphate (saturated solution) is α = 54.42%.

Up to pH = 4.5, the separation degree of zinc from the solution increases sharply. Between pH = 4.5 and 8, the separation degree is at its maximum, and at pH > 8, the separation degree decreases. The optimal pH value of the reaction mass can be considered to be pH = 6–7, which leads to the formation of a filtrable and washable precipitate.

- Molar ratio NH_3_:Zn^2+^.

**Figure 4:**
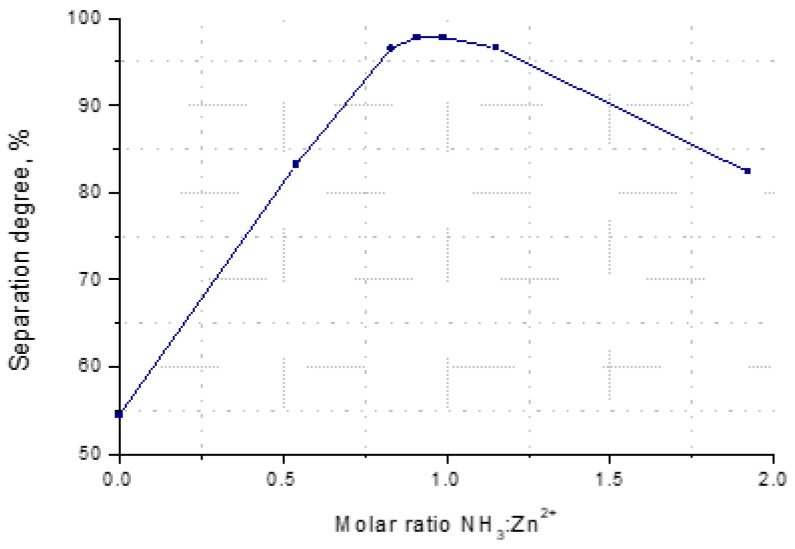
Dependence of the separation degree of zinc (α) from the solution of molar ratio of NH_3_:Zn^2+^, for a solution of 265 g/L Zn^2+^, at 25 °C.

Zinc’s maximum separation degree from the solution is determined by a molar ratio of NH_3_:Zn^2+^ = (0.83–0.99):1 (Figure 4).

- Zinc concentration.

**Figure 5:**
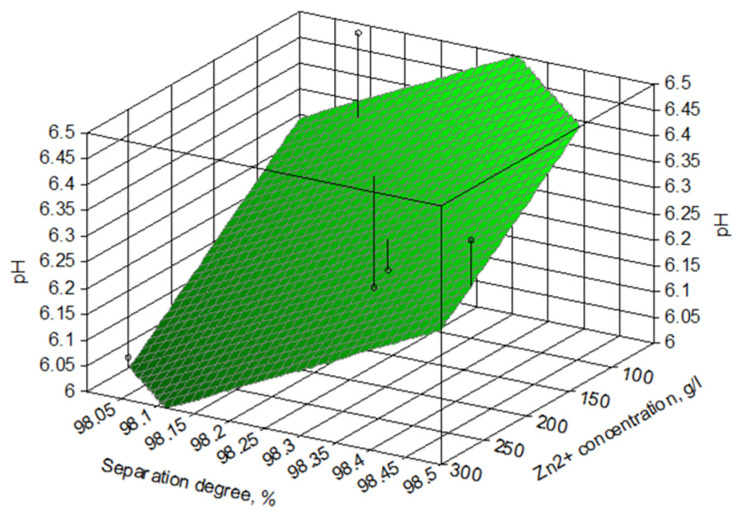
Dependence of the separation degree of zinc (α) of the solution on zinc concentration, for a solution of 265 g/L Zn^2+^ at optimal pH.

The experimental data on the influence of Zn^2+^ concentration in the solution on the separation degree of zinc are shown in Figure 5.

The experimental data show that at optimal pH (pH ≈ 6) and a temperature of 25 °C, the separation degree of zinc from the solution is maximum, regardless of the concentration of zinc in the solution (within the studied limits).

- Temperature.

**Figure 6:**
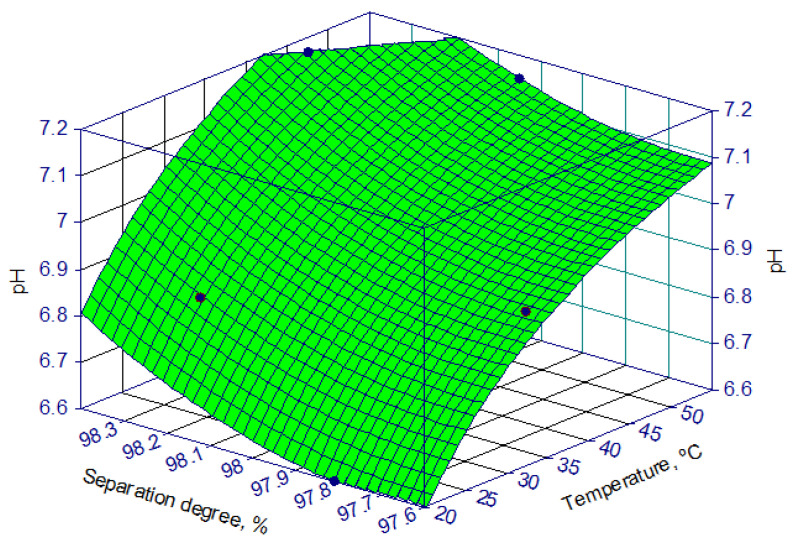
Dependence of the separation degree of zinc (α) on temperature for a solution of 265 g/L Zn^2+^ at optimal pH.

The experimental data on the influence of temperature on the separation degree of zinc from the solution are presented in Figure 6.

From these data, it results that, in optimal pH conditions, the separation degree of zinc from the solution is not practically influenced by the temperature (within the studied limits). This study shows that the optimal temperature for the zinc–ammonium phosphate obtaining process is approximately 45 °C.

- Duration of the process.

The experimental research has shown that, under optimal pH conditions, to obtain a crystalline precipitate that is easy to filter, the process duration is approximately 50–60 min.

**Figure 7:**
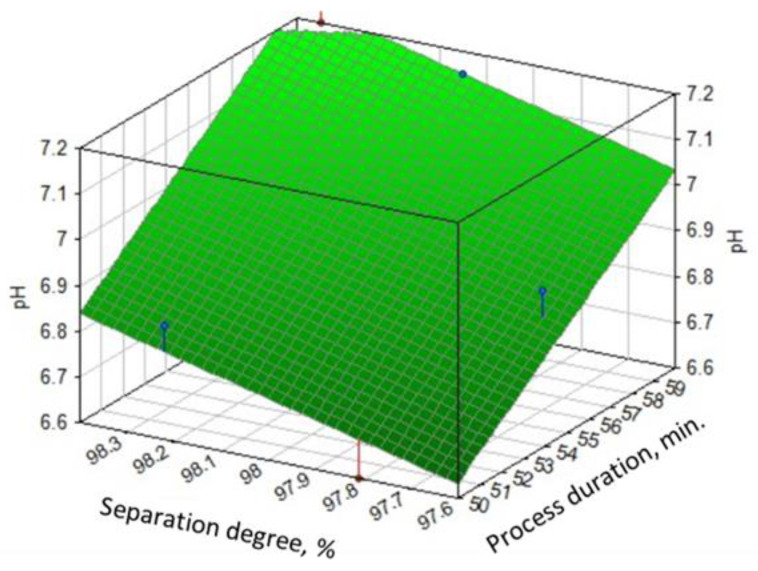
Dependence of the separation degree of zinc (α) on the process duration for a solution of 265 g/L Zn^2+^ at optimal pH.

The experimental data regarding the influence of the process duration on the separation degree of zinc from the solution at optimal pH are presented in Figure 7.

In conclusion, the optimal conditions for the processing of zinc chloride solutions with diammonium phosphate and ammonia, which determine a maximum separation degree of zinc (α ≈ 98%), and the formation of a crystalline precipitate that settles and filters easily are a molar ratio of (NH_4_)_2_HPO_4_:Zn^2+^ = 1.02:1, pH of the reaction mass pH ≈ 6–7, temperature of 45 °C and a process duration of 50–60 min.

### 3.3. Studies on the Obtained Products

#### 3.3.1. Chemical Composition

The precipitate obtained by adding diammonium phosphate and neutralizing with an ammonia solution of the solution resulting from the dissolution of the sludge with 20% HCl with a concentration of 265 g/L Zn^2+^ was separated by filtration, and then the concentration of Zn, N and P_2_O_5_ was determined.

**Figure 8:**
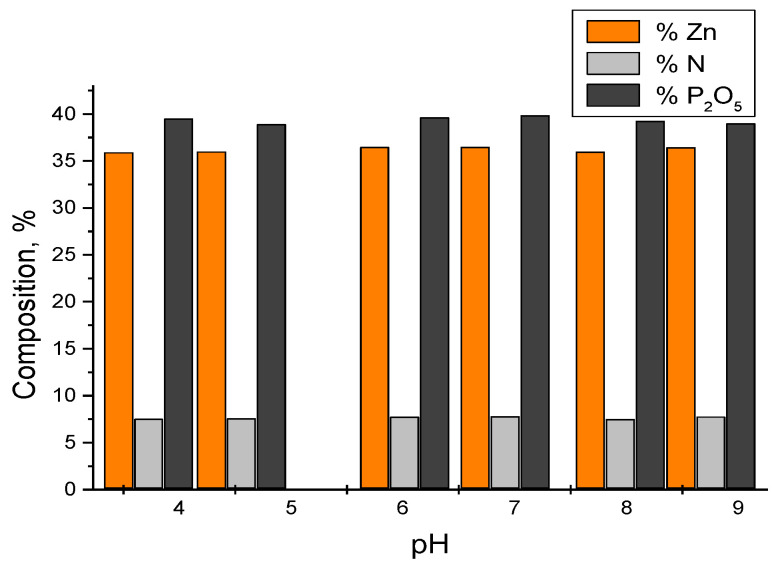
Composition of products obtained from solutions with a content of 265 g/L Zn^2+^ at 25 °C at different values of the pH of the reaction mass.

The experimental data obtained are presented in Figure 8.

According to these experimental data, the products obtained in the processing of zinc chloride solutions with diammonium phosphate and ammonia have, as a basic component, zinc–ammonium phosphate ZnNH_4_PO_4_.

From the experimental data, the possibility of the extraction of metal ions and their valorization in the form of complex phosphates with microelements, with uses in technique or as fertilizers with microelements, is observed [1,23,24].

#### 3.3.2. X-ray Diffraction Studies

The obtained products were subjected to X-ray diffraction analysis. X-ray diffraction was measured with a Shimadzu-600 diffractometer using CuKα radiation [18,19].

**Figure 9:**
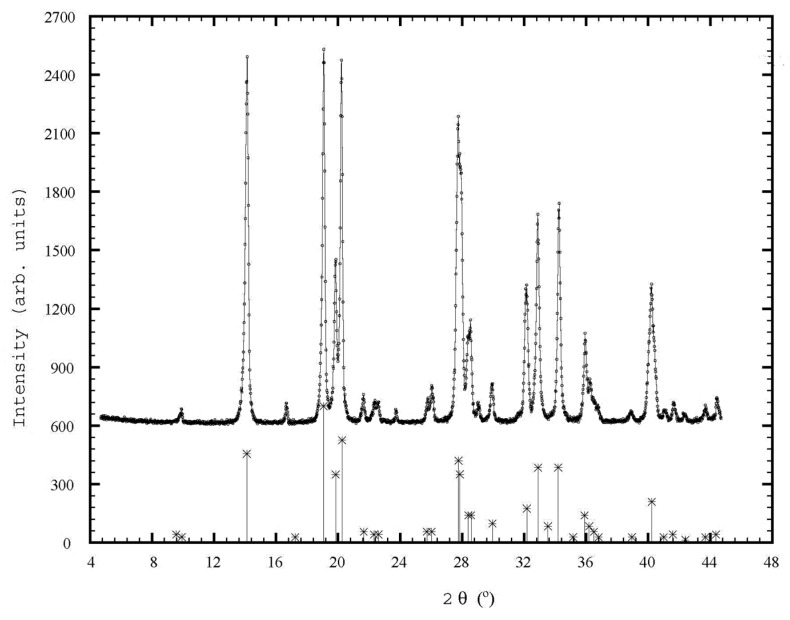
Diffractogram of product obtained at pH ≈ 6.6, temperature 45 °C.

A diffractogram of the product obtained at the molar ratio (NH_4_)_2_HPO_4_:Zn^2+^ = 1.02:1, pH of the reaction mass pH ≈ 6.6 and temperature of 45 °C is shown in Figure 9.

The model matches the standard one for ZnNH_4_PO_4_ in the JCPDF 22-0025 database. By indexing the data in the diffractogram, it is observed that the product crystallizes in an orthorhombic system, with the following parameters: a = 8.796 Å, b = 5.456 Å, c = 8.965 Å and the volume of the unit cell V = 430,239 Å^3^.

A comparison of the diffractogram with those from the literature [27,28,29] confirms that the product obtained has, as a main component, zinc–ammonium phosphate ZnNH_4_PO_4_.

#### 3.3.3. FT-IR Spectroscopic Studies

FT-IR spectroscopic investigations were performed with a JASCO 6100 spectrometer in the spectral range of 4000–400 cm^−1^ with a resolution of 2 cm^−1^ using the KBr pelleting technique [25,26].

**Figure 10:**
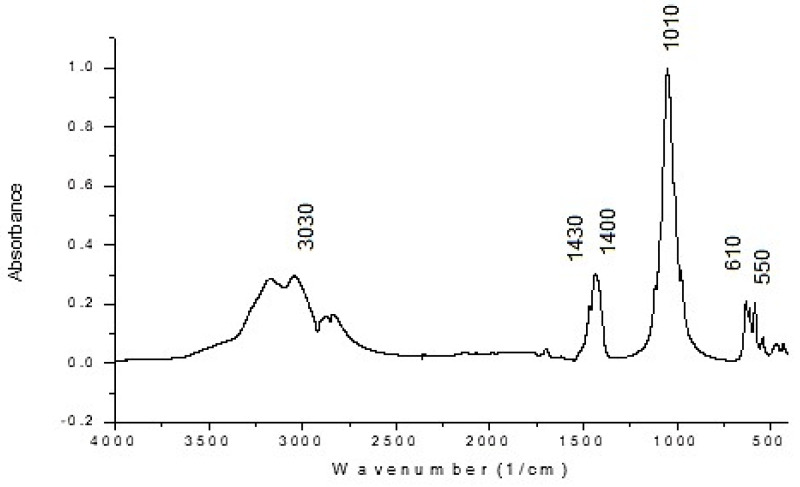
FT-IR spectrum for product obtained at pH ≈ 6.6, temperature 45 °C.

With the FT-IR spectrum, the frequency ranges of bands characteristic of the PO_4_^3−^, NH_4_^+^ ion groups were established. The FT-IR spectrum for the product obtained under optimal conditions (molar ratio of (NH_4_)_2_HPO_4_:Zn^2+^ = 1.02:1, pH of the reaction mass ≈ 6.6, temperature of 45 °C) is shown in Figure 10.

The FT-IR spectrum shown in Figure 10 reflects the characteristic stretching patterns of NH_4_^+^, PO_4_^3−^. The absorption observed at 3030 and 1400–1430 cm^−1^ is attributed to the N–H stretching mode of NH_4_^+^. The combining of water stretching and N–H stretching produces a broad peak between 3400 and 2900 cm^−1^. The absorption peak at 1010 cm^−1^ is assigned to the PO_4_^3−^ asymmetric stretching mode and the sharp absorption peak at 970 cm^−1^ is assigned to the PO_4_^3−^ symmetric stretching mode. The strong absorption bands at 610–550 cm^−1^ are attributed to PO_4_^3−^ bending vibrations mode [1,23,24,29].

Comparing the results obtained with those from the specialized literature [29,30,31,32] confirms that the product obtained by treating zinc chloride solutions with diammonium phosphate and ammonia corresponds to zinc–ammonium phosphate ZnNH_4_PO_4_.

#### 3.3.4. Thermogravimetric and Thermodifferential Studies

**Figure 11:**
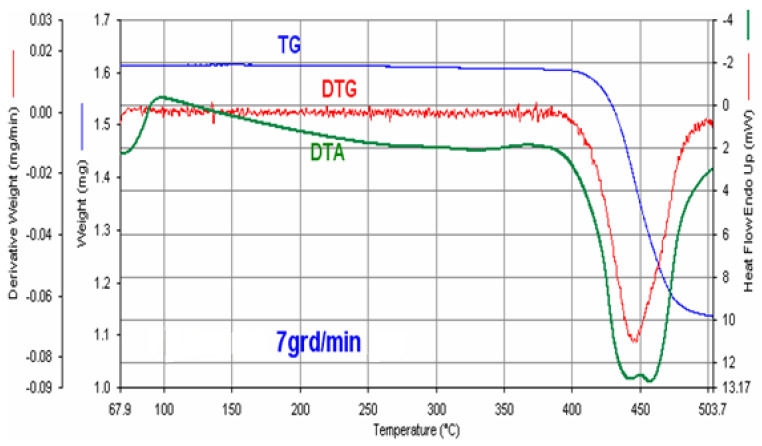
Thermogravimetric and thermodifferential curves for product obtained at pH ≈ 6.6, temperature 45 °C.

In order to determine some physicochemical properties, thermal stability and composition, the product obtained under optimal conditions was subjected to thermal and thermogravimetric studies (Figure 11).

The process takes place in three stages. The first stage, in the temperature range of 100–400 °C, is characterized by relatively small mass losses caused by the removal of moisture and part of the ammonia contained. The second stage, in the temperature range of 400–460 °C, is characterized by significant mass losses of about 15% caused by the total elimination of ammonia and the start of the process of converting phosphate into pyrophosphate. The third step, in the temperature range of 460–550 °C, is characterized by a slow rate of decomposition determined by the completion of phosphate transformation into zinc pyrophosphate.

The thermal effects occurring at 420 °C and 475 °C correspond to the elimination of ammonia from zinc–ammonium phosphate transforming zinc–ammonium phosphate into zinc pyrophosphate. Thus, thermal and thermogravimetric studies show that the obtained product corresponds to zinc–ammonium phosphate ZnNH_4_PO_4_ [30,32].

#### 3.3.5. Scanning Electron Microscopy (SEM) Studies

**Figure 12:**
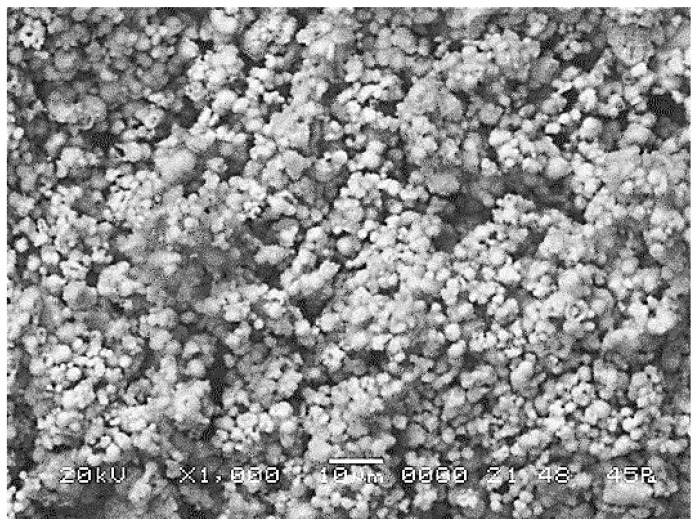
SEM appearance of a ZnNH_4_PO_4_ granule.

**Figure 13:**
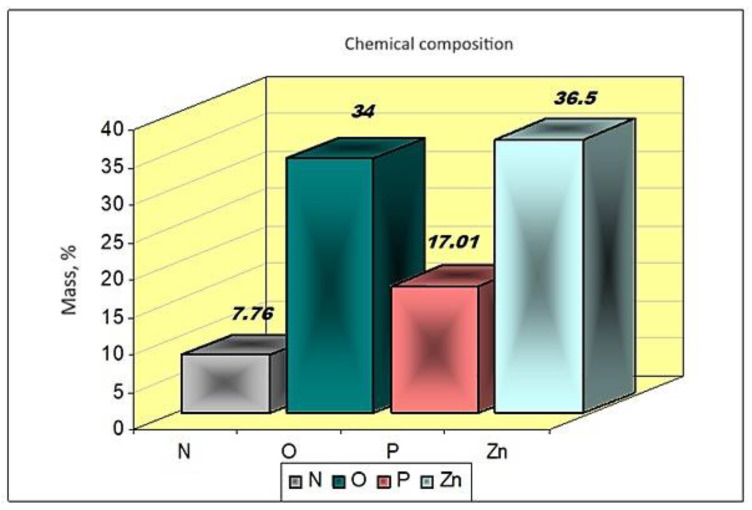
Chemical composition diagram of a ZnNH_4_PO_4_ granule.

**Figure 14:**
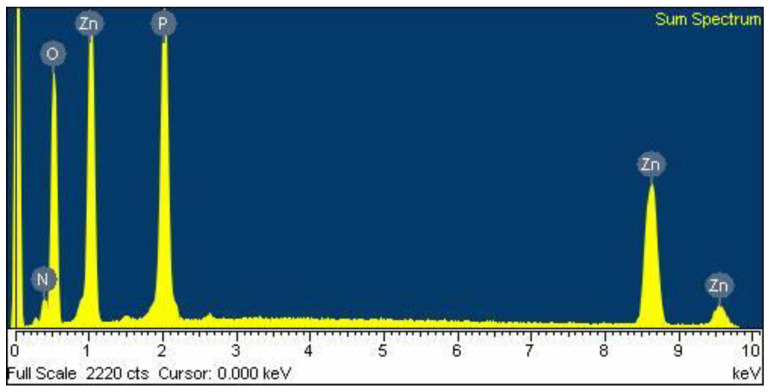
EDS (X-ray) spectra of the elements in the analyzed micro area.

Figure 12, Figure 13 and Figure 14 show the appearance of a granule in the product under analysis, the diagram of the chemical composition of a granule and the EDS spectrum (of X-rays) of the elements on the analyzed micro area.

With electronic scanning microscopy, the appearance of the granular surfaces, the X-ray spectra showing the presence of the elements on the analyzed micro area and the diagram of the chemical composition on the granule of each synthesized compound are observed.

In the case of zinc–ammonium phosphate, it is observed that the particles are irregular and agglomerated, in which the spherical shape predominates, with submicron dimensions. Particle morphology provides a relatively high degree of packaging in the case of press compaction. The analyzed segment is 10 μm and the magnification is 1000× [33,34,35,36,37].

## 4. Conclusions

This present paper aims to extract zinc ions from waste and reevaluate them in the form of zinc–ammonium phosphate. Initially, the sludge from the acid zinc galvanizing baths was analyzed to determine its composition. The metal ions were extracted from the sludge by dissolving it in hydrochloric acid solutions of varying concentrations. It was observed that the concentration of acid used directly affected the amount of zinc ions extracted. To produce zinc–ammonium phosphate, the most concentrated solution of zinc ions was utilized. This solution was obtained by dissolving the sludge in a hydrochloric acid solution with a concentration of 20%. Diammonium phosphate and a 25% ammonia solution were used to obtain zinc–ammonium phosphate. The variation of the pH of the reaction mass was followed by the mass and molar ratio of NH_3_:Zn^2+^.

The optimal conditions for the process of processing zinc chloride solutions with diammonium phosphate and ammonia, which determine a maximum separation degree of zinc (α ≈ 98%), and in which a crystalline precipitate that settles and filters easily is formed, are a molar ratio of (NH_4_)_2_HPO_4_:Zn^2+^ = 1.02:1, pH of the reaction mass pH ≈ 6–7, temperature of 45 °C and a process duration of 50–60 min.

Studies on chemical and phase composition, RX studies, FT-IR analysis, thermogravimetric analysis and SEM show that the products obtained while processing zinc chloride solutions (the most concentrated solution of zinc ions, 265 g/L Zn^2+^) with diammonium phosphate and ammonia correspond to ZnNH_4_PO_4_ zinc–ammonium phosphate.

In conclusion, this study demonstrates the successful utilization of residual sludge from acid zinc electroplating baths as a viable source of zinc for obtaining zinc–ammonium phosphate. This process not only showcases environmental protection by repurposing industrial waste but also contributes to more sustainable practices in the production of zinc–ammonium phosphate.

## Figures and Tables

**Table 1:** Composition of the sludge used.

Element	Composition, %
K	0.75 ± 0.01
Na	0.37 ± 0.01
Cu	0.06 ± 0.005
Fe	5.7 ± 0.01
Zn	65.5 ± 0.05
Cr	bdl

## Data Availability

All data generated/used in this study are presented in the published manuscript.
